# Abstracts of Papers Read at the International Medical Congress

**Published:** 1881-10-20

**Authors:** 


### ABSTRACTS OF PAPERS READ AT THE INTERNATIONAL MEDICAL CONGRESS.

---

*The Curability of Uterine Displacements, by* Paul F. Munde, M.D., *New York.*—Finding that the text-books either entirely omitted all mention of the possibility of permanently curing displacements of the uterus by any of the methods in use, or gave but vague statements on the subject, and impressed with the importance of having some positive conclusions on this matter, both for the sake of the patient and the satisfaction of the physician, the author had analyzed the cases of displacement which had come under his care (895), and had arrived at the following conclusions :

1. Displacements of the uterus are permanently curable in the large majority of cases only when recent, or when a complete tissue metamorphosis, as occurs during pregnancy and after parturition, takes place.

2. Chronic cases (of more than a year's standing) are but rarely curable permanently, except occasionally under the last named circumstances. Apparent cures reported by some authors and witnessed by many physicians, soon show themselves to have been but temporary.

3. Pessaries form unquestionably the most practical, rational and (temporarily) the most efficient means of treating uterine displacements. Cures are but rarely accomplished by them.

4. Medicated (chiefly astringent) tampons, intelligently applied every day by the physician, give the best chances for permanent cure. This is particularly true of prolapsus, but holds good for all forms.

5. Electricity locally applied deserves more extended application.

6. All methods should be persevered in for months and years before success is to be expected.

*Total Extirpation of the Uterus, by* William Freund, M.D., *Strasburg.* —The author said that his experience of three years, which had elapsed since its first publication on total extirpation of the uterus, and the works of others on the same subject which had appeared within this interval, had rendered his judgment clear as regards the most weighty points in the matter. There could be no question that, in carcinomatous disease of the uterus, extending over a considerable portion of this organ, total extirpation was the operation indicated. Some time ago surgeons repeatedly recurred to this operation, and tried both the operation through the vagina and that through incision of the abdominal walls. The results of these attempts were so unfortunate, that for a long time no further were made. When Dr. Freund had put his method to the test, with a favorable result in the first case; and when the first cases operated upon by Dr. Martini in Breslau, Dr. Kochs in Bonn, Prof. Olshausen in Halle, Prof. Schroder in Berlin, Dr. Velt in Berlin, Prof. Spiegelberg in Breslau, Dr. Kuhn in St. Gallen, had likewise proved successful, the hope, and even a somewhat excessive confidence, that he had at last safely solved this great problem of surgery. was intelligible and justified. But when unfavorable results were frightfully multiplied, when, also, the mournful fact became clear that recurrences did occur, discouragement and opposition appeared on many sides. Greater safety to life, according to most recent experience, was secured by the total extirpation *per vaginam*, carried out according to the principles of Czerny, Billroth, Schroder, Martini, and the abdominal total extirpation, modified according to the directions of Bardenheuer, Breisky, Rydygier, Kolaczek, and M. B. Fruend. The leaving open and drainage of the peritoneal cavity, the simple ligaturing of the vessels of the severed broad ligaments, step by step, made the operation shorter, less laborious, and hastened the healing. Dr. Freund had convinced himself by experiment upon the dead body that, after separation of the cervix uteri from the vault of the vagina, it was easy to draw up the uterus through the abdominal wound above the symphysis. Dr. Rydygier and the author had carried out this mode of operating, first recommended by Breisky: By drawing up the uterus by the tenaculum forceps invented by Dr. Freund, the uterus was rendered at once comparatively bloodless. The severing of the cervix all round from the vagina, he had carried out, without chloroforming the patient, immediately before the actual operation. The results of the vaginal total extirpation, as regards recovery, appeared to be very good; and Kolaczek affirmed that, in the method of abdominal total extirpation, as practiced by him and Martini, a fatal result was exceptional. The operation might be undertaken as a not very dangerous one in the early stages of carcinoma and sarcoma, in which it gave a promise of radical curc. Whether the vaginal or the abdominal extirpation was to be performed, must be decided according to the individual case. If the uterus were very large, and the vagina very narrow, the abdominal total extirpation must always be undertaken. With a small uterus and capacious vagina the vaginal operation was to be preferred: but the abdominal operation had a great advantage, in facilitating and insuring the carrying out of the separation of the uterus through sound tissue. On July 5th, Dr. Freund extirpated the uterus by the abdominal method, in spite of the uterus having been small and the vagina sufficiently large, in order to remove several cancerous intra-abdominal (iliac) glands. Advanced knowledge had shown that the danger of bleeding was not so great as was once supposed; and the danger of the peritoneal aperture was no longer to be considered—nay, rather, that the keeping open of the peritoneal wound was highly desirable.

*Antisepsis in midwifery, by* O. Spiegelberg, M.D., *Breslau.*—The author said that the great reform in surgery brought about by the antiseptic treatment could not fail to have a deep influence upon the treatment of the complications in childbed, as it was well known long ago that the latter were the same as arose from wounds. If, however, scrupulous cleanliness, which had been advocated long ago, favored a normal course of the puerperium, the practical gain was not very great. The idea that puerperal wounds were infected, and that inflammation of the genital organs was initiated by germs coming from outside, became more in vogue; and the idea that phlogogenous matter might be produced spontaneously within the genital tract was almost abandoned. The consequence of this idea was reeommending the most scrupulous cleanliness of hands and instruments; forbidding students engaged in dissecting to attend midwifery cases; forbidding nurses attending cases of puerperal fever to attend normal cases at the same time. The experience that all these measures only slightly reduced the number of bad cases, originated the idea of secondary antisepsis. Intra-uterine irrigations and drainage came into use, but without much avail; the opinion took root that there existed an essential puerperal process. The application of the theory and practice of Lister's system to the puerperium meant the strictest cleanliness and antisepsis during birth, both on the part of the persons attending the mother, and of the mother herself. Air must be prevented from entering the genital tract; and as that was not wholly unavoidable, disinfection by frequent irrigation with antiseptics during birth may be practiced. After birth, care must be taken to secure perfect rest for the genital tract, to encourage involution, avoiding every intra-vaginal or intra-uterine manipulation which was not absolutely necessary; if such were necessary, it must be done under strictly antiseptic precautions. Secondary antisepsis—after the infection had taken place—was not of much avail. It was only directly useful in processes of decomposition, so long as they had not passed the surface of the tract, and had not yet attacked the parenchyma of the organs. Otherwise, antisepsis was only a palliative, but no trustworthy remedy, since drainage and irrigation did not hit the deep seat of the disease, and did not remove or destroy the entered germs, not to speak of the inconveniences of the practical application of the secondary antisepsis.—*Med. and Surg. Reporter.*

